# Comparing accuracy of bedside ultrasound examination with physical examination for detection of pleural effusion

**DOI:** 10.1186/s13089-021-00241-7

**Published:** 2021-09-06

**Authors:** Michael H. Walsh, Kang X. Zhang, Emily J. Cox, Justin M. Chen, Nicholas G. Cowley, Christopher J. Oleynick, Leo M. Smyth, Irene W. Y. Ma

**Affiliations:** 1 Division of General Internal Medicine, University of Calgary, Calgary, AB Canada; 2 Providence Health Care, Providence Internal Medicine Residency Spokane, Spokane, WA USA; 3 Providence Health Care, Providence Medical Research Center, Spokane, WA USA; 4 Division of General Internal Medicine, University of Alberta, Edmonton, AB Canada; 5 Department of Medicine, W21C, University of Calgary, 3330 Hospital Dr NW, Calgary, AB T2N 4N1 Canada; 6 Department of Community Health Sciences, W21C, University of Calgary, Calgary, AB Canada

**Keywords:** Ultrasound, Point-of-care ultrasound, Pleural effusion, Physical examination

## Abstract

**Background:**

In detecting pleural effusion, bedside ultrasound (US) has been shown to be more accurate than auscultation. However, US has not been previously compared to the comprehensive physical examination. This study seeks to compare the accuracy of physical examination with bedside US in detecting pleural effusion.

**Methods:**

This study included a convenience sample of 34 medical inpatients from Calgary, Canada and Spokane, USA, with chest imaging performed within 24 h of recruitment. Imaging results served as the reference standard for pleural effusion. All patients underwent a comprehensive lung physical examination and a bedside US examination by two researchers blinded to the imaging results.

**Results:**

Physical examination was less accurate than US (sensitivity of 44.0% [95% confidence interval (CI) 30.0–58.8%], specificity 88.9% (95% CI 65.3–98.6%), positive likelihood (LR) 3.96 (95% CI 1.03–15.18), negative LR 0.63 (95% CI 0.47–0.85) for physical examination; sensitivity 98% (95% CI 89.4–100%), specificity 94.4% (95% CI 72.7–99.9%), positive LR 17.6 (95% CI 2.6–118.6), negative LR 0.02 (95% CI 0.00–0.15) for US). The percentage of examinations rated with a confidence level of 4 or higher (out of 5) was higher for US (85% of the seated US examination and 94% of the supine US examination, compared to 35% of the PE, *P* < 0.001), and took less time to perform (*P* < 0.0001).

**Conclusions:**

US examination for pleural effusion was more accurate than the physical examination, conferred higher confidence, and required less time to complete.

## Background

Pleural effusions are common in general medical patients and may be caused by pathological states such as congestive heart failure, infections, cirrhosis, and malignancy [1, 2]. Detection of pleural effusions is important because their presence may signal a need for diagnostic and/or therapeutic interventions [2, 3]. Traditionally in internal medicine, bedside identification of pleural effusions involves performing a physical examination (PE), followed by imaging studies [4]. PE includes a number of maneuvers, with dullness to percussion and asymmetric chest expansion considered the most accurate signs [5, 6].

One study found that with minimal training, novice residents were able to reliably detect pleural effusions using bedside ultrasound (US) [7]. In contrast, only 60% of medical residents accurately detected bronchial breath sounds [8], such as those that might occur above the level of a pleural effusion [9], with no appreciable increase in auscultation accuracy between 1st year and 3rd year residents [8]. While studies suggest that US outperforms PE for detecting pleural effusion [10–14], many studies used only auscultation as the comparator, rather than a comprehensive PE. Thus, the superior performance of US in these studies may be exaggerated. To address this gap, our study seeks to compare the diagnostic accuracy of a multi-component PE with that of bedside US in detecting pleural effusions in medical patients.

## Methods

The Conjoint Health Research Ethics Board at the University of Calgary and the Providence St. Joseph Health Institutional Review Board approved this study. This study is reported to conform to STARD guidelines for reporting studies of diagnostic accuracy [15].

### Aim

This study seeks to compare the diagnostic accuracy of a PE with that of bedside US in medical inpatients.

### Design and setting

A convenience sample of consenting patients were prospectively recruited from the Foothills Medical Centre (Calgary, AB, Canada) between August 2019 and March 2020 and Providence Sacred Heart Medical Center (Spokane, WA, USA) between September 2019 and June 2020. Patients admitted to the general medical ward who had chest computed tomography (CT) or chest radiography (CXR) performed within 24 h of the study period were eligible. Exclusion criteria were hemodynamic instability, inability to comply with the study protocol, or presence of pain/dressings that would preclude an US scan. Recruitment occurred when patient permission to approach the patient was provided by the admission team and when at least two researchers were available.

Consenting patients underwent both a PE and a bedside US examination independently by two researchers. The order of the examinations and the researcher performing each examination were randomized.

### Physical examination (PE) protocol

With the patient in a sitting position, the researcher inspected the posterior thorax for asymmetry, and performed percussion, tactile fremitus, auscultation, and egophony. Based on these findings, the researcher recorded the PE diagnosis and rated their confidence in the diagnosis using a 5-point Likert scale, where 1 = not at all confident and 5 = very confident. This procedure was then repeated on the other side by the same researcher, and time to complete the examination was recorded.

### Ultrasound examination protocol

**Fig. 1:**
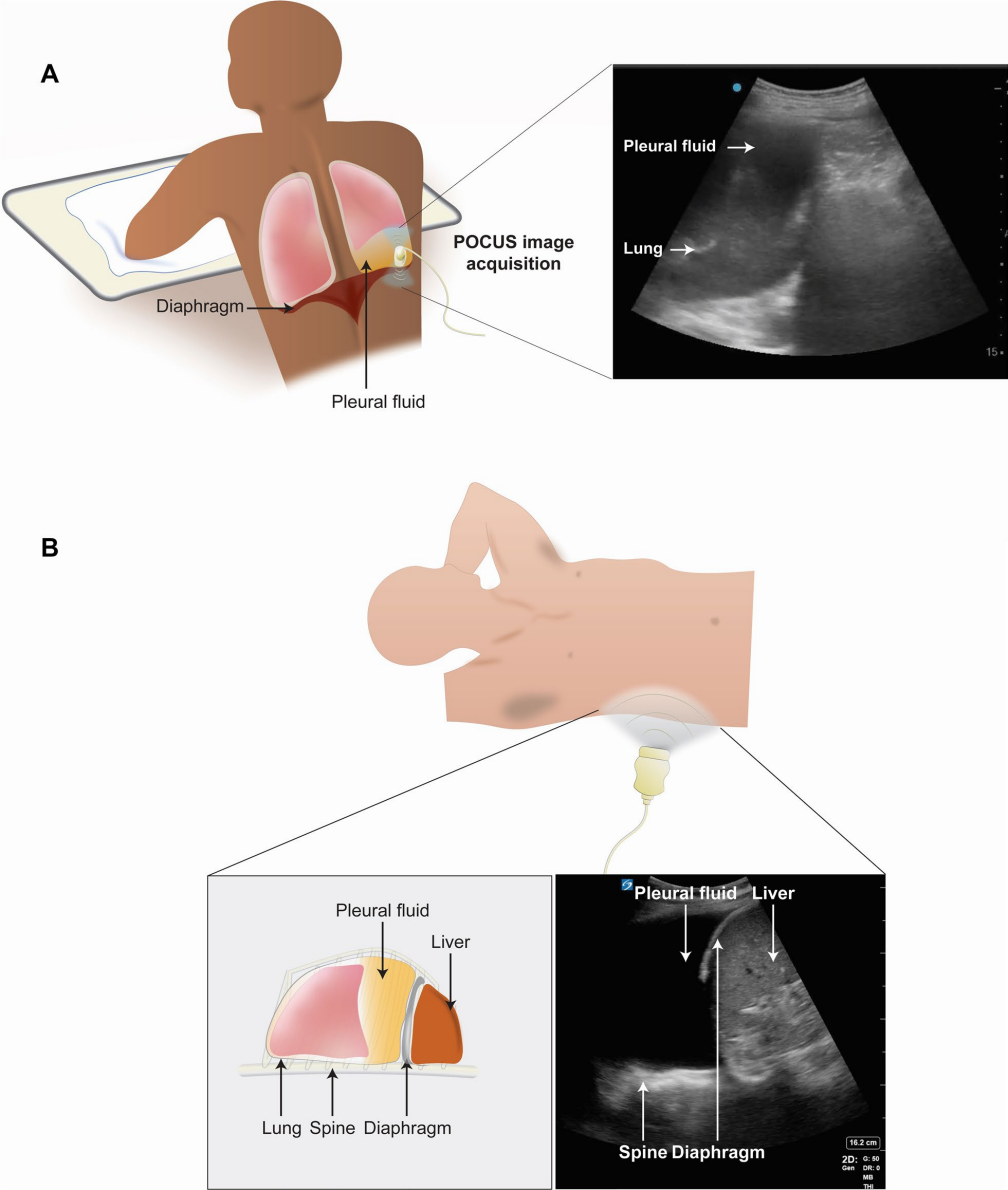
Procedure for detecting pleural effusion with ultrasound. To detect the presence or absence of pleural effusion with ultrasound, the patient is evaluated in the seated position (**A**) and the supine position (**B**). With the patient in the seated position (**A**), the researcher slides the longitudinally oriented transducer inferiorly from the lung apices. With the patient in the supine position (**B**), the researcher obtains a coronal view of the lung base. A pleural effusion is detected if the researcher identifies both a positive spine sign and free fluid

A different researcher, blinded to the PE results, performed the US exam. With the patient in the seated position, a low-frequency transducer (2–5 MHz curvilinear array, Edge II, in Calgary; 1–5 MHz phased array, M-Turbo, Sonosite Inc., in Spokane) was used to longitudinally scan the posterior thorax inferiorly from the lung apices (Fig. 1A). The diagnosis was noted and the procedure was repeated on the other side.

Next, with the patient in a supine position, the same researcher performed coronal views of the lower lung zones (Fig. 1B). The presence of the spine sign and free fluid was used to determine the presence of pleural effusion. Time taken to complete the US examination in the seated and supine position and self-reported confidence were recorded.

### Reference standard

Chest imaging findings reported by radiologists at each institution on chart review served as the reference standard for the diagnosis of pleural effusion. All researchers were blinded to the imaging study results at the time of the PE and US examinations.

### Researcher training

Three researchers performed the study protocol in Calgary. Two were certified in Internal Medicine by the Royal College of Physicians and Surgeons of Canada and completing their point-of-care ultrasound fellowships (MW, LS). The third (CO) was a post-graduate year (PGY-3) medical resident who underwent a 1-h didactic session on US pleural effusion, and 6 h of supervised lung US scanning prior to patient enrollment. Of the PEs, five were completed by researcher MW, eight by CO, and four by LS. Of the US examinations, 11 were performed by MW, three by CO, and three by LS.

At the Spokane site, two researchers conducted the study protocol: one was a PGY-3 medical resident (NC), who completed eight PEs and nine US examinations for this study protocol, and the other was a PGY-4 clinical teaching fellow (JC), who completed nine PEs and eight US examinations for this study. Both completed a 4-h didactic session on bedside US and 12 h of supervised lung US scanning during a 2-week bedside US elective prior to patient enrollment.

### Study outcomes

The primary outcomes were the diagnostic accuracies of PE and US. Secondary outcomes included time required to perform the examinations and overall confidence.

### Statistical analyses

Based on the previously reported pooled sensitivity of 0.93 for US [16], assuming an alpha of 0.5, and a prevalence of 0.94, a sample size of 28 would be required for our study [17, 18]. We used Wilcoxon rank-sum tests, Fisher exact tests, analysis of variance, and post hoc Tukey tests (where appropriate) to compare differences between groups. The diagnostic accuracy of the examinations was compared to the reference standard using sensitivity, specificity, positive likelihood ratio (LR) and negative LR. LRs greater than 10 or less than 0.1 were considered large effects; LRs of 5 to 10 and 0.1 to 0.2 moderate; 2 to 5 and 0.5 to 0.2 small; 1 to 2 and 0.5 to 1 negligible [19]. Confidence between groups was compared using Kruskal–Wallis tests and Fisher’s exact tests adjusted for multiple comparisons using Bonferroni corrections. All analyses were performed using SAS version 9.4 (SAS Institute Inc., Cary, NC).

## Results

A convenience sample of 34 patients (*n* = 17 from Calgary and *n* = 17 from Spokane) consented to participate (Table 1). Based on chest imaging, 22 (65%) had bilateral pleural effusions, six (27%) had unilateral, and six (27%) had no effusions. Twenty patients had a CXR performed, two had a CT performed, and 12 had both CT and CXRs performed. For those with both imaging studies performed, there was complete concordance in the pleural effusion diagnosis. There was no missing data nor known adverse events from either the reference imaging studies nor the physical examination or bedside US studies.

**Table 1:**
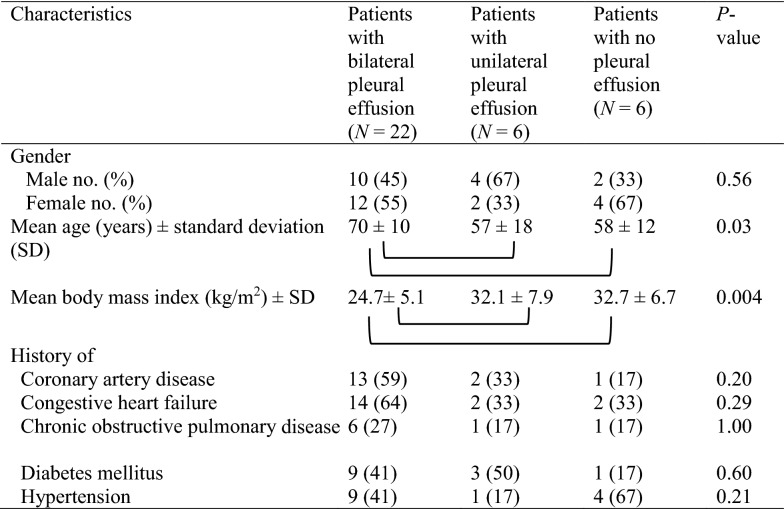
Baseline patient characteristics of the 34 patients included in the study

Square brackets indicate subgroups that are statistically different from each other (*P* < 0.05) in post hoc analyses

### Physical exam

Decreased tactile fremitus and egophony were more commonly noted in cases of pleural effusion (Table 2). The overall PE for pleural effusion had a sensitivity of 44.0% and specificity of 88.9% (Table 3). Chest asymmetry had the highest specificity (94.4%) while dullness to percussion had the highest sensitivity (94.0%).

**Table 2:** Physical examination and bedside ultrasound findings in 34 patients

	All lungs (*N* = 68 lungs)	Lungs with pleural effusion (*N* = 50 lungs)	Lungs with no pleural effusion (*N* = 18 lungs)	*P*-value
**Physical examination findings**				
Decreased tactile fremitus—No. (%)	37 (54)	34 (68)	3 (17)	0.0002
Crackles—No (%)	30 (44)	23 (46)	7 (39)	0.78
Bronchial breath sounds—No. (%)	53 (78)	39 (78)	14 (78)	1.00
Dullness to percussion—No. (%)	62 (91)	47 (94)	15 (83)	0.33
Asymmetric chest wall expansion—No. (%)	10 (15)	9 (18)	1 (6)	0.27
Egophony—No. (%)	21 (31)	19 (38)	2 (11)	0.04
PE diagnosis—No. (%)				
Normal/diaphragm	41 (60)	25 (50)	16 (89)	0.005
Pleural effusion	24 (35)	22 (44)	2 (11)	0.02
Other (consolidation/atelectasis)	10 (15)	8 (16)	2 (11)	1.00
Median level of confidence (interquartile range) out of 5	3 (2–4)	3 (1–4)	3.5 (2–4)	0.04
Not at all confident—No. (%)	14 (21)	13 (26)	1 (6)	0.09
Somewhat not confident—No. (%)	14 (21)	10 (20)	4 (22)	
Neutral—No. (%)	16 (24)	12 (24)	4 (22)	
Somewhat confident—No. (%)	22 (32)	15 (30)	7 (39)	
Very confident—No. (%)	2 (3)	0	2 (11)	
**Bedside ultrasound (US) findings**				
**Seated, posterior US exam**				
Ultrasound diagnosis as pleural effusion	47 (69%)	46 (92)	1 (6)	< 0.0001
Median level of confidence of seated, posterior US exam (interquartile range) out of 5	5 (4–5)	5 (5–5)	5 (4–5)	0.10
Not at all confident—No. (%)	5 (7)	4 (8)	1 (6)	0.15
Somewhat not confident—No. (%)	0	0	0	
Neutral—No. (%)	0	0	0	
Somewhat confident—No. (%)	10 (15)	5 (1)	5 (28)	
Very confident—No. (%)	48 (71)	38 (76)	10 (56)	
Missing responses	5 (7)	3 (6)	2 (11)	
**Supine, coronal view US exam**				
Ultrasound diagnosis as pleural effusion (positive spine sign)	50 (74%)	49 (98)	1 (6)	< 0.0001
Median level of confidence of supine US exam (interquartile range) out of 5	5 (5–5)	5 (5–5)	5 (4–5)	0.09
Not at all confident—No. (%)	0	0	0	0.12
Somewhat not confident—No. (%)	1 (1)	0	1 (6)	
Neutral—No. (%)	1 (1)	1 (2)	0	
Somewhat confident—No. (%)	11 (17)	6 (12)	5 (28)	
Very confident—No. (%)	53 (78)	41 (82)	12 (67)	
Missing responses—No. (%)	2 (3)	2 (4)	0	

Findings are presented in terms of the individual count of lungs included in the study (*N* = 68 lungs from 34 patients)

**Table 3:** Diagnostic performance of physical examination and lung ultrasound for detection of pleural effusion

	Sensitivity (95% CI)	Specificity (95% CI)	Positive likelihood ratio (95% CI)	Negative likelihood ratio (95% CI)	Accuracy (95% CI)
Overall physical examination	44.0% (30.0–58.8%)	88.9% (65.3–98.6%)	3.96 (1.03–15.18)	0.63 (0.47–0.85)	55.9% (44.3–67.9%)
Individual physical examination findings					
Decreased tactile fremitus	68.0% (53.3–80.5%)	83.3% (58.6–96.2%)	4.08 (1.43–11.7)	0.38 (0.24–0.60)	72.1% (59–9-82.3%)
Dullness to percussion	94.0% (83.5–98.8%)	16.7% (3.6–41.4%)	1.13 (0.91–1.40)	0.36 (0.08–1.62)	73.5% (61.4–83.5%)
Asymmetric chest wall expansion	18% (8.6–31.4%)	94.4% (72.7–99.9%)	3.24 (0.44–23.81)	0.87 (0.73–1.03)	38.2% (26.7–50.8%)
Egophony	38.8% (25.2–53.8%)	88.9 (65.3–98.6%)	3.49 (0.90–13.50)	0.69 (0.52–0.91)	52.2% (39.7–64.6%)
Bedside ultrasound examination					
Seated (posterior exam)	92% (80.8–97.8%)	94.4% (72.7–99.9%)	16.6 (2.5–111.4)	0.08 (0.03–0.22)	92.7% (83.7–97.6%)
Supine (coronal views)	98% (89.4–100%)	94.4 (72.7–99.9%)	17.6 (2.6–118.6)	0.02 (0.00–0.15)	97.1% (89.8–99.6%)

Diagnostic parameters and their 95% confidence intervals (CI) of physical examination and sonographic findings for pleural effusion

### US exam

Both the seated and supine US examination had high diagnostic accuracies, (sensitivity 92% and 98%, specificity 94.4% and 94.4%, respectively for seated and supine US exam, Table 3).

### Secondary outcome measures

Mean duration of the PE [2 min 53 s ± standard deviation (SD) 39 s] was longer than the US exam in the seated and supine position (2 min 6 s ± SD 38 s and 1 min 37 s ± 43 s, respectively, *P* < 0.05 for each comparison, Table 2). For confidence in the PE diagnosis, 24 (35%) of the 68 examinations were rated with a score of four or higher (out of five), while 58 (85%) of the US seated examination and 64 (94%) of the US supine examinations were rated as four or higher (Table 2). After accounting for multiple comparisons, where a *P* < 0.017 is needed for statistical significance, post hoc analyses revealed that confidence in the PE was significantly lower than either of the US examinations (*P* < 0.001 for both), while the confidence between the two US examinations was not significantly different (*P* = 0.27).

### Discussion

In our study of general medical inpatients, bedside US examination demonstrated higher accuracy than the PE. Sitting and supine US examinations resulted in diagnostic LRs that are considered large in magnitude, while LRs associated with PE were small [19]. Additionally, US examinations resulted in higher confidence and took less time to perform. While asymmetric chest wall expansion and dullness to percussion demonstrated high specificity and high sensitivity, respectively, the associated LRs were negligible to small [19].

Our supine US examination results (sensitivity of 98%, specificity 94.4%, a diagnostic accuracy of 97.1%) are consistent with existing literature. A pooled sensitivity of 93% and specificity of 96% were noted in a prior systematic review [16], while a diagnostic accuracy of 95.1% was previously reported [20]. In contrast, for dullness to percussion, our positive LR 1.13 is lower than the previously reported LR of 8.7, although a wide 95% CI (2.2–33.8) was noted [5]. Pooled negative LR of 0.31 in that review was similar to ours (0.36) [5]. Also consistent with existing literature was the finding that asymmetric chest expansion demonstrated a favorable specificity of over 90% [5, 21]. However, our positive LR was 3.24, while theirs was 8.1 [5], which may be a function of protocol differences, such as having trainees perform PE in our study, rather than experienced clinicians [5, 21]. However, even with experienced clinicians, their positive LR of 8.1 was only moderate in strength.

Few studies directly compared PE with bedside US for pleural effusion. Five prior studies reported that US was superior [10–14]. However, in at least four of these studies, only auscultation was performed [11–14], and auscultation is known to be less accurate than other PE maneuvers [5, 6]. In another study, added to PE, US resulted in significantly higher odds of identifying pleural effusion compared to PE alone [22]. Our present study adds to existing literature by directly comparing US with a comprehensive PE. Our results demonstrate that, despite the high specificity associated with asymmetric chest wall expansion and high sensitivity for dullness to percussion, the small likelihood ratios associated with these PE maneuvers suggest that US should be the preferred approach. Further, the associated large likelihood ratios for US suggest that, in the hands of a trained POCUS practitioner, the presence of pleural effusion can likely be ruled in or ruled out, with reasonably high accuracy, especially if the supine examination was used, evaluating for the presence or absence of the spine sign.

Our study has some limitations. First, our study sample had very few controls; only 6% did not have a pleural effusion. Although patients were not selected based upon the results of chest imaging studies, we cannot rule out the possibility that permission by the admitting team to approach patients for the study was more likely when the patient had pleural effusion, thereby introducing a potential selection bias. The resultant high prevalence of pleural effusions in our study sample is important to note for two reasons. First, representativeness of our conclusions may be limited, thereby limiting generalizability of our study conclusions [19]. Secondly, a high prevalence may also influence our resultant diagnostic accuracies, given the restriction in range seen as well as high reader expectations [23]. However, prior studies have reported that lower specificity may be reported in studies with higher prevalence [23]. Second, because of the multiple examinations required of our volunteer patients for this study protocol and the need for two clinician researchers present per patient, we were not able to perform an additional examination by an independent researcher. Thus, we do not have inter-rater reliability data for our study. Prior studies suggest that inter-rater reliability for US examination is at least moderate or higher for pleural effusions [7, 24]. Third, our reported US duration did not include the time required for the machine to be located, transported to the bedside, and subsequently turned on. Thus, the actual time required may vary in the real-world setting. Fourth, resident-performed examinations may be less accurate than those performed by board-certified internists. However, senior trainees at both institutions commonly perform PEs on admission. Fifth, we did not collect information on subsequent patient management decisions, any ensuing procedures, or patient preferences regarding PE vs. US. In addition, we did not collect information on the admission diagnosis. At the Calgary site, the most common medical admission diagnoses were: congestive heart failure, alcohol withdrawal, type 1 diabetes mellitus with ketoacidosis, pneumonia, and fluid/electrolyte/acid base disorders. At the Spokane site, the most common admission diagnoses were: sepsis, acute respiratory failure, cerebral infarction, hypertensive heart and kidney disease, and alcoholic liver disease. Finally, the majority of our reference standards were based on CXR results. CT would be a preferred gold standard. However, sensitivity analysis of our diagnostic parameters did not result in significant changes to our conclusions when limiting our analyses to only those with CT results (*data not shown*). In detecting pleural effusion, US may be more sensitive than CXR [16, 25, 26], and could be identifying clinically insignificant effusions. However, US can directly visualize septations and complex effusions, and in that regard, has a theoretical advantage over CXR and CTs [27]. Neither of these outcomes were examined in our study, but should be considered in future studies.

## Conclusions

In conclusion, US examination for pleural effusion is more accurate, confers greater confidence, and may be quicker to complete than PE. Thus, in situations where the device is readily accessible and practitioner is trained to scan and interpret, US examination for pleural effusion would be preferred.

## Data Availability

The datasets used and/or analyzed during the current study are available from the corresponding author on reasonable request.

## Abbreviations

CXR: Chest radiography
CT: Computed tomography
CI: Confidence interval
IQR: Interquartile range
LR: Likelihood ratio
PE: Physical examination
PGY: Post-graduate year
SD: Standard deviation
US: Ultrasound

## References

[1] Beaudoin S, Gonzalez AV (2018). Evaluation of the patient with pleural effusion. CMAJ.

[2] Hooper C, Lee YCG, Maskell N (2010). Investigation of a unilateral pleural effusion in adults: British Thoracic Society Pleural Disease guideline 2010. Thorax.

[3] Light RW (2011). Pleural effusions. Med Clin N Am.

[4] Saguil A, Wyrick K, Hallgren J (2014). Diagnostic approach to pleural effusion. Am Fam Physician.

[5] Wong CL, Holroyd-Leduc J, Straus SE (2009). Does this patient have a pleural effusion?. JAMA.

[6] Mcgee S (2018). Evidence-based physical diagnosis.

[7] Begot E, Grumann A, Duvoid T (2014). Ultrasonographic identification and semiquantitative assessment of unloculated pleural effusions in critically ill patients by residents after a focused training. Intensive Care Med.

[8] Mangione S, Nieman LZ (1999). Pulmonary auscultatory skills during training in internal medicine and family practice. Am J Respir Crit Care Med.

[9] Sarkar M, Madabhavi I, Niranjan N, Dogra M (2015). Auscultation of the respiratory system. Ann Thorac Med.

[10] Patterson LA, Costantino TG, Satz WA (2004). Diagnosing pleural effusion: a prospective comparison of physical examination with bedside thoracic ultrasonography. Ann Emerg Med.

[11] Lichtenstein D, Goldstein I, Mourgeon E, Cluzel P, Grenier P, Roubyj J (2004). Comparative diagnostic performances of auscultation, chest radiography, and lung ultrasonography in acute respiratory distress syndrome. Anesthesiology.

[12] Vezzani A, Manca T, Brusasco C (2014). Diagnostic value of chest ultrasound after cardiac surgery: a comparison with chest x-ray and auscultation. J Cardiothorac Vasc Anesth.

[13] Tasci O, Hatipoglu ON, Cagli B, Ermis V (2016). Sonography of the chest using linear-array versus sector transducers: correlation with auscultation, chest radiography, and computed tomography. J Clin Ultrasound.

[14] Inglis AJ, Nalos M, Sue KH (2016). Bedside lung ultrasound, mobile radiography and physical examination: a comparative analysis of diagnostic tools in the critically ill. Crit Care Resusc.

[15] Cohen JF, Korevaar DA, Altman DG (2016). Stard 2015 guidelines for reporting diagnostic accuracy studies: explanation and elaboration. BMJ Open.

[16] Grimberg A, Shigueoka DC, AAtallahn AN, Ajzen S, Iared W (2010). Diagnostic accuracy of sonography for pleural effusion: systematic review. Sao Paulo Med J.

[17] Truc TT (2020) Statistics and sample size pro. https://Play.Google.Com/Store/Apps/Details?Id=Thaithanhtruc.Info.Sass&Hl=En_Us&Gl=Us. Accessed 21 July 2021.

[18] Negida A, Fahim NK, Negida Y (2019). Sample size calculation guide—part 4: how to calculate the sample size for a diagnostic test accuracy study based on sensitivity, specificity, and the area under the roc curve. Adv J Emerg Med.

[19] Guyatt G, Rennie D, Meade M, Cook D (2015). Users' guides to the medical literature: a manual for evidence-based clinical practice.

[20] Xie C, Sun K, You Y (2020). Feasibility and efficacy of lung ultrasound to investigate pulmonary complications in patients who developed postoperative hypoxaemia-a prospective study. BMC Anesthesiol.

[21] Kalantri S, Joshi R, Lokhande T (2007). Accuracy and reliability of physical signs in the diagnosis of pleural effusion. Respir Med.

[22] Steinmetz P, Oleskevich S, Dyachenko A, Mccusker J, Lewis J (2018). Accuracy of medical students in detecting pleural effusion using lung ultrasound as an adjunct to the physical examination. J Ultrasound Med.

[23] Leeflang MMG, Rutjes AWS, Reitsma JB, Hooft L, Bossuyt PMM (2013). Variation of a test's sensitivity and specificity with disease prevalence. CMAJ.

[24] Kumar A, Weng Y, Graglia S (2021). Interobserver agreement of lung ultrasound findings of Covid-19. J Ultrasound Med.

[25] Hansell L, Milross M, Delaney A, Tian DH, Ntoumenopoulos G (2021). Lung ultrasound has greater accuracy than conventional respiratory assessment tools for the diagnosis of pleural effusion, lung consolidation and collapse: a systematic review. J Physiother.

[26] Yousefifard M, Baikpour M, Ghelichkhani P (2016). Screening performance characteristic of ultrasonography and radiography in detection of pleural effusion: a meta-analysis. Emergency (Tehran).

[27] Svigals PZ, Chopra A, Ravenel JG, Nietert PJ, Huggins JT (2017). The accuracy of pleural ultrasonography in diagnosing complicated parapneumonic pleural effusions. Thorax.

